# Parental responses to child experiences of trauma following presentation at emergency departments: a qualitative study

**DOI:** 10.1136/bmjopen-2016-012944

**Published:** 2016-11-07

**Authors:** Victoria Williamson, Cathy Creswell, Ian Butler, Hope Christie, Sarah L Halligan

**Affiliations:** 1 Department of Psychology, University of Bath, Bath, UK; 2 Department of Psychology, University of Reading, Reading, UK; 3 Department of Psychiatry, University of Cape Town, South Africa

**Keywords:** QUALITATIVE RESEARCH

## Abstract

**Objective:**

Parents are often children's main source of support following fear-inducing traumatic events, yet little is known about how parents provide that support. The aim of this study was to examine parents' experiences of supporting their child following child trauma exposure and presentation at an emergency department (ED).

**Design:**

Semistructured qualitative interviews analysed using thematic analysis.

**Setting:**

The setting for this study was two National Health Service EDs in England.

**Participants:**

20 parents whose child experienced a traumatic event and attended an ED between August 2014 and October 2015.

**Results:**

Parents were sensitive to their child's distress and offered reassurance and support for their child to resume normal activities. However, parental beliefs often inhibited children's reinstatement of pretrauma routines. Support often focused on preventing future illness or injury, reflective of parents' concerns for their child's physical well-being. In a minority of parents, appraisals of problematic care from EDs contributed to parents' anxiety and perceptions of their child as vulnerable post-trauma. Forgetting the trauma and avoidance of discussion were encouraged as coping strategies to prevent further distress. Parents highlighted their need for further guidance and support regarding their child's physical and emotional recovery.

**Conclusions:**

This study provides insight into the experiences of and challenges faced by parents in supporting their child following trauma exposure. Perceptions of their child's physical vulnerability and treatment influenced parents' responses and the supportive strategies employed. These findings may enable clinicians to generate meaningful advice for parents following child attendance at EDs post-trauma.

**Strengths and limitations of this study**

- The children included in this study were exposed to a broad range of traumatic events which allows for the inclusion of a wide range of parental views and experiences.
- Reliability of the qualitative analysis was confirmed by the independent assessment of all transcripts, codes and themes by an additional qualitative researcher for agreement. Participants were provided with a summary of the interview findings to ensure the validity and the robustness of the findings.
- Child trauma exposure was limited to single-incident, physical trauma and may not reflect the experiences of parents of children exposed to chronic trauma or trauma not associated with significant physical consequences.
- The majority of participating parents were mothers and father/other caregiver views were less well represented.
- Parent–child dyads were recruited from a relatively low-risk, Western context based in England which may not be generalisable to other contexts without further investigation.

## Introduction

Traumatic events, such as serious road traffic accidents or accidental injury, are relatively common in childhood^1^ ^2^ and are associated with a range of psychological adjustment difficulties, including post-traumatic stress disorder (PTSD)^3^ ^4^ which can result in long-term adverse outcomes.^5^ ^6^ Parents are often children's leading source of support post-trauma, and parental behaviours may mediate children's resilience.^7^ Previous research has shown that parental warm support following child trauma exposure is associated with fewer child PTSD symptoms.^8–10^ Conversely, parental overprotection and advocacy of avoidant coping may increase the risk of child PTSD symptoms.^11–13^ Such post-trauma parental responses are likely to be influenced by parents own psychopathology and distress.^7^ ^14^ ^15^ Notably, child trauma exposure can cause parental post-traumatic distress, even when parents were not directly exposed to the event,^16^ and parental post-trauma distress may result in the promotion of maladaptive coping strategies or parental difficulties in discussing the trauma, which can negatively impact child adjustment.^17^ ^18^

Given the interplay between child trauma/PTSD, parental distress and parental support, it is striking that few studies have considered how parents experience supporting children post-trauma, what challenges they face or the factors that influence their approach.^15^ ^19^ One notable qualitative study, conducted in the Netherlands, provided evidence that parents can be proactive in responding to child post-traumatic distress, taking steps to ensure that they are aware of their child's needs and support their child accordingly.^19^ Nonetheless, parental experiences following child trauma exposure remain underexplored. A deeper understanding of parents' experiences of caring for a child post-trauma may inform clinical practice and contribute to the development of meaningful and acceptable guidance for families in which a child has experienced trauma.

We conducted in-depth, qualitative interviews with 20 parents following their child's attendance at hospital emergency departments (EDs) in England. We aimed to explore parents' perceptions of (need for) support, the experiences of parents in supporting their child post-trauma and the impact of child trauma on family processes.

## Method

The study received approval from the National Health Service Research Ethics Committee (14/SC/0043) and Bath University Ethics Committee (15-218). Participants gave informed consent (parents) or assent (children).

### Participants

Twenty parents and their children were recruited following the child's attendance at one of two EDs in the south of England. Recruitment took place between August 2014 and October 2015. Participants were eligible for the study if the child was aged 6–16 years and had experienced a traumatic event as defined by DSM-V criterion A for PTSD.^20^ The following exclusion criteria were applied: parent or child inability to speak English; child organic brain damage or intellectual disability that precludes mainstream schooling; child registered with child protection services and concerns that the respondent parent inflicted the trauma.

Of the 53 eligible patients approached by the clinical care team, 33 declined (ie, 37% recruitment rate, consistent with other ED studies^21^). Reasons for decline as reported to the clinical care team included fatigue and ‘wanting to put the event behind them’.

### Assessments

#### Parental PTSD symptoms

Parents completed the 49-item Post-traumatic Stress Diagnostic Scale (PDS)^22^ as a measure of their own PTSD symptoms. Symptom items are rated on a 4-point Likert scale (total score range 0–51) and the scale has good test–retest reliability and internal consistency.^23^

#### Child PTSD symptoms

Child PTSD was measured by the University of California at Los Angeles (UCLA) Post-traumatic Stress Disorder Reaction Index,^24^ a widely used measure of child post-traumatic stress symptoms (PTSS) with good internal consistency and test–retest reliability.^25^ The University of California at Los Angeles Posttraumatic Stress Disorder Reaction Index (UCLA-RI) is based on the DSM-IV PTSD criteria, has parent and child report versions and indexes trauma exposure as well as symptoms. Symptom frequencies are rated on five-point Likert scales ranging from 0 (“never”) to 4 (“most of the time”), with symptoms scored as present if rated 3 (‘much of the time’) or greater. If criterion A is met, children who meet criteria B, C and D are given a likely ‘full’ diagnosis of PTSD, and children who meet criteria for only two symptom subcategories are given a ‘partial’ PTSD diagnosis.^25^ All parents completed the parent report version of the UCLA-RI in relation to their child's recent experience. In addition, children completed symptom scales (part 2) of the UCLA-RI child report version,^24^ providing their own reports of PTSS relating to the event that led to their ED admission. Full information, based on parent and child reports, is presented in table 2.

### Qualitative interview schedule and procedure

Interviews were conducted by a female doctoral student (VW) who had training and experience in qualitative methods. Interviews were conducted by telephone and lasted 57 min on average (range=23.5–92.6 min). The researcher did not have a relationship with participants prior to study initiation. We developed the interview topic guide based on the research questions and the literature on parent–child trauma recovery responses. Interview questions focused on parents' post-trauma responses, concerns about their child and experiences of providing support (see online supplementary file 1). Parents were also asked for their views on the support available post-trauma. Interviews were audio-recorded and transcribed verbatim. Twenty parents completed the qualitative interview, and thematic saturation was achieved.

10.1136/bmjopen-2016-012944.supp1 supplementary data

As respondent validation, we provided parents with a written summary of the key findings and preliminary interpretations following the interview. This opportunity to obtain participant feedback further increased the potential reliability and accuracy of the data.^26^ In fact, only three parents responded to correct factual details which did not alter the thematic analysis. We treated input from participants regarding the interview summary as additional data.

### Procedure

Purposive sampling was used. Participants were initially identified by the clinical care team following ED attendance. The clinical team sought parental permission for their details to be passed to the research team. Given this agreement, parents were contacted by the study researcher by telephone with further information about the study. Following informed consent/assent, participating parents and children first completed assessments of their psychological adjustment and then parents participated in the qualitative interviews. Parents and children were approached by the clinical care team on average 2 weeks following ED attendance, and families were recruited to the study ∼4 weeks post-trauma (range=10–98 days).

### Data analysis

We used NVivo V.10 (http://www.qsrinternational.com/products_nvivo.aspx) to conduct thematic analysis on participant transcripts.^27^ We used the steps proposed by Braun and Clarke:^27^ reading and re-reading the data, generating initial codes, searching for and developing candidate themes and revising and classifying themes. An inductive analytic approach was used, with initial codes and themes proposed by VW. A reflexive journal was kept throughout data collection and analysis by the primary researcher (VW) in an effort to recognise the influence of the researcher's prior experiences, thoughts and assumptions and prevent premature or biased interpretations of the data. To ensure reliability, all transcripts, codes and themes were independently reviewed by authors VW and HC. Disagreements between authors were infrequent and were resolved following discussion and re-examination of the data. Peer debriefing was conducted, and feedback regarding data interpretation and analysis was sought from coauthors IB and SLH.

## Results

### Descriptive information

Of our final sample, 75% of participating parents were mothers, 40% of the participating children were female with a mean age of 10.4 years (SD=3.2) (see table 1). The average UCLA-RI parent-report score was 9.6 (SD=10.3, Mdn=6), and the average UCLA-RI child-report score was 10.73 (SD=7.4, Mdn=10). Three children were classified as having a likely PTSD diagnosis using the UCLA-RI, and three children met criteria for a partial diagnosis. The mean PDS score was 7.9 (SD=8.9, Mdn=7) which is considered mild.^28^ Trauma characteristics are described in table 2.

**Table 1:** Participant information

Index	Sample statistics (n=20)
Child mean age, M (SD)	10.4 (3.2)
Child female gender, n (%)	8 (40%)
Parent mean age, M (SD)	41.6 (6.1)
Parent marital status, n (%)	
Single	1 (5%)
Married (first time)/cohabiting	17 (85%)
Remarried	2 (10%)
Mean time since trauma, M(SD)	41 days (26.2) (Mdn=32.5)
Trauma types	
RTA	8 (40%)
Assault	1 (5%)
Fall from elevation	5 (25%)
Acute medical emergency	4 (20%)
Sporting injury	1 (5%)
Other	1 (5%)
Percentage of children admitted as inpatient, n (%)	14 (70%)
Mean number of injuries sustained, M (SD)	1.95 (1.7)
Mean income, n (%)	
Don't wish to respond	4 (20%)
<£10 000	1 (5%)
£10 000–29 000	3 (15%)
£30 000–49 000	2 (10%)
£50 000–69 000	5 (25%)
£70 000–200 000	5 (25%)
Ethnicity, n (%)	
White British	15 (75%)
Black British	1 (5%)
Asian British	2 (10%)
Other	2 (10%)

UCLA-RI, parent and child report, PTSD overall severity score reported. Number of children meeting criteria for PTSD diagnosis refers to the number of children meeting criteria for a PTSD diagnosis based on their responses on the UCLA-RI. Mean time since trauma=mean number of days post-trauma at the time of the interview.

M, mean; Mdn, median; PTSD, post-traumatic stress disorder; RTA, road traffic accident; UCLA-RI, UCLA Post-traumatic Stress Disorder Reaction Index.

**Table 2:** Participant trauma characteristics

ID no.	Child age	Child gender	Parent interviewed	Parent age	Parent involved in the event	Trauma experienced	Days since trauma	UCLA-RI Severity Score (parent report)	UCLA-RI Severity Score (child report)	PDS Symptom Severity Score
A	6	Female	Mother	25	Yes	Acute medical emergency	18	20*,†	0	15
B	8	Female	Father	42	No	RTA	32	1	18	7
C	15	Male	Father	42	No	Near-drowning	69	5	6	1
D	14	Female	Mother	45	No	Fall from elevation	38	6	23‡	22
F	6	Male	Mother	40	Yes	Acute medical emergency	92	6	NA	8
G	11	Female	Mother	45	No	RTA	33	4	18	19
H	7	Female	Father	49	No	Fall from elevation	25	4	6	0
I	16	Male	Mother	45	No	Assault	48	42*	10	22
J	9	Male	Mother	40	No	RTA	55	7	20‡	9
K	11	Male	Mother	44	No	RTA	23	17	20	NA
L	13	Male	Mother	29	No	RTA	10	17	20*,†	7
M	8	Female	Mother	42	No	Fall from elevation	80	6	10	0
N	14	Male	Mother	42	No	RTA	25	4	6	0
O	12	Female	Mother	42	Yes	Acute medical emergency	17	8	13	1
P	9	Male	Father	44	No	RTA	28	27†,‡	13	27
Q	11	Male	Father	50	Yes	Sports injury	23	7	2	0
R	6	Male	Mother	41	Yes	RTA	98	5	14	4
S	10	Male	Mother	37	No	Fall from elevation	53	2	4	0
T	9	Female	Mother	49	Yes	Fall from elevation	11	0	0	0
U	13	Male	Mother	39	No	Acute medical emergency	42	3	3	9

UCLA-RI, parent and child report. PTSD overall severity score reported. PDS, parent symptom severity score reported. Parent involved in the event refers to whether or not the parent was directly involved in or witnessed the child's traumatic event.

*Meets criteria for likely PTSD diagnosis using UCLA-RI.

†Time since trauma less than 4 weeks therefore duration criterion cannot be applied in this case. NA, data unavailable as parent did not complete or refused for child to take part.

‡Meets criteria for partial PTSD diagnosis using UCLA-RI.

PDS, Post-traumatic Stress Diagnostic Scale; PTSD, post-traumatic stress disorder; RTA, road traffic accident; UCLA-RI, UCLA Post-traumatic Stress Disorder Reaction Index.

### Qualitative results

Five key themes emerged from the data reflecting parents' experiences and attempts to support their child post-trauma. Anonymised participant comments are provided to illustrate our findings, and all participants have been assigned a pseudonym.

#### Post-trauma perception of the child and event

Parents described several changes in their child's behaviour following the trauma and understood many of these changes to be a result of their child's distress following the experience of trauma.

PID K: He did quite like going out on his own… But he's a bit scared now…doesn't wanna cross any roads on his own…the day that we actually left hospital he was really scared of crossing the roads straightaway…he's holding my hand like it was vice like grip.

Some parents were unconcerned by these changes in their child as their post-trauma anxiety was considered to be a result of and limited to the trauma (eg, fear of water after near-drowning). Other parents described their child as essentially unchanged, with any behavioural changes attributed to their physical injuries post-trauma.

Interviewer: Were there any changes that you noticed in Ian after his accident?

PID S: He has quietened down a little bit…he's been in a lot more [playing] computer games but…its more because he physically can't join in, not because…it mentally affected his personality or that he's worried.

Parents often compared their child's post-trauma behaviour to their pretrauma behaviour to determine whether their child was coping. Parents understood their children to be coping well when they exhibited no behavioural changes or when pretrauma activities were resumed. Children were also considered to be coping if they did not talk or ask questions about the trauma. Parents thought their child not ruminating about or dwelling on the event contributed to their capacity to cope.

PID S: He's not worried about anything…it's not like he's coming with questions “what if?” or “why did this happen to me?”…he doesn't have any of those kind of feelings or fears, he knows it's a few weeks and hopefully it will heal.

#### Strategies to support the child

Parents' experiences of the trauma and their child's subsequent medical care influenced the support they provided. The most prominent themes in parents' narratives reflected a desire to care for their child post-trauma and for family life to continue as normal, while protecting children from potential future harm.

*Warm support*: Parents reported making themselves available for their child and encouraging them to talk to them about their post-trauma distress. Discussion of the event and associated distress were thought to be instrumental to the child's recovery, and some parents engaged in lengthy discussions to facilitate their child's disclosure of their feelings.

PID K: I'm very much “tell mummy how you feel?” sort of thing… It's important to for them to tell you how they're feeling emotionally…so I think in that way, because of the way we are, I think that's helped a lot really.

Children experienced significant anxiety post-trauma and parents responded with reassurance that the event would not reoccur and normalisation of their post-trauma distress. Parents attempted to address their child's anxiety by initiating confidence building exercises and being nearby to offer reassurance in fear-provoking situations. Parents advocated a positive interpretation of the trauma by positively reframing the event and encouraging children to feel lucky as the event could have been worse.

PID K: We're gonna do a little bit of road safety… I said to him… “I'm gonna be you…and you're gonna be the parent and we're gonna cross the road together” and he said “what happens if I get you run over?” and I said “you won't get me run over dear.”

Despite these supportive strategies, parents also described considerable helplessness in caring for their child, particularly during lengthy hospital stays as their child required medical attention that they personally could not provide. To manage feelings of helplessness, parents tried to be actively involved in their children's medical treatment and after-care, for example by purchasing medical equipment to monitor their child's health at home.

PID F: [A friend] told me about this pixel meter…[so] I went to see a nurse…and she gave it to us… I feel like I have at least something to measure if he needs more oxygen or not, so I feel like at least I have something because when he got home from hospital I was thinking well how would I know?

*Returning to normal:* Parents attempted to continue their family's pretrauma routines to encourage their child's emotional and physical recovery through physical activity. Accommodating children's post-trauma difficulties and distress was often time limited, and parents gradually encouraged children to resume their normal activities. Notably, parents simultaneously struggled to reinstate pretrauma routines because of their own anxiety that their child may experience future harm, as seen in the following section.

PID I: [We're] just trying to be normal and try and not to baby him too much, to sort of try and encourage him to do things a bit more on his own but not wanting to push it too much, you know, it's still sort of quite soon after.

Encouragement of trauma-related discussions was not universal, and some parents instead advocated cognitive and behavioural avoidant coping strategies. Parents removed their child from contact with trauma reminders, which were thought to hinder recovery, and encouraged children to forget the event.

PID R: It was very difficult for me because I didn't want to upset him in one way, he'd already been hurt… I did say “why did you let go of mummy's hand?” and after that I didn't ask [that was] the only one time I asked… I said to him “now be a child and try to forget about it…what has happened, happened, let's move on from it.”

Parents reported avoiding discussion of the trauma to prevent their child becoming distressed. Discussion of the trauma was thought to be unnecessary and potentially harmful as it would prevent their child moving on from the event or strengthen their trauma memories.

PID B: I don't think she talk about [it], she did not talk a lot about the accident and I don't want to ask her either, I'm afraid that will brought back some terrible memory, so I did not ask her.

In these circumstances, if the trauma was discussed it was performed in a factual, perfunctory manner, with conversation focused on the child's physical recovery. As a result, some parents were unaware whether their child was experiencing post-trauma distress.

PID F: He ask me like “Mummy is it better to live or die?”…but I think with me I was a bit upset so I didn't really talk about like “how do you feel Daniel?” Well I did ask him like “are you OK?” but I didn't really emotionally ask it…and I didn't really want to remind him too much of it afterwards…we wanted to just carry on as normal and not to get him upset or worried too much.

One assumption held by parents was that their child would feel able to initiate a discussion of the event and their associated distress if needed, despite parental avoidance of trauma-related discussions. If children did not broach the subject, they were considered to be coping well.

PID Q: I would say he's just moved on… he's not going on about it…it's not as if were sitting down to [ask] “are you OK after your accident?” and all that stuff…and he is the sort of boy who if it was on his mind would talk to us.

*Guarding:* Children were considered vulnerable post-trauma and parents expressed significant concerns that their child could re-experience serious illness or injury. Parents were vigilant of their child's physical symptoms, such as headaches and breathlessness, and encouraged others to be observant of symptoms.

It was particularly difficult for parents to be apart from their child post-trauma, which contributed to their struggle to resume their family's pretrauma routines. Parents implemented significant changes to their child's daily routine to prevent future illness or injury. Children were described as persuading their parents to allow them to resume activities parents now considered risky, and parents coped with their anxiety by checking on children frequently.

PID D: I'm still worried, you know, I think I drove her mad really, sort of following her round and saying “you can't do this, you can't do that, you know be careful with what you're doing, do you need pain killers?”…I'm finding it difficult to let her do things that she did before without worrying…[and] it was just difficult to have her away from me really.

#### Perceptions and impact of medical treatment

Parents largely reported that their child had received good quality medical treatment from EDs and were treated quickly and professionally. Where present, perceived problems in medical care, including misdiagnosis and limited or insensitive communication about medical procedures, contributed to parents' anxiety and perceptions of children as vulnerable and needing future protection.

PID D: We were told, which was actually incorrect, that she didn't have any bleeds on the brain… I think that happening has made me more nervous because I'm thinking “well if they've got something wrong once, you know, they could be wrong again or it could be worse than we're thinking”.

On discharge, parents reported not receiving information about their child's physical recovery and it was difficult to access follow-up appointments to confirm their progress. This also contributed to parental anxiety and feelings of helplessness as parents felt uncertain of what to expect during their child's recovery and/or of whether symptoms were normal. Ideally, parents would have preferred to receive information about their child's physical recovery in-person by a doctor on discharge; information from leaflets or the internet was not considered useful.

PID L: We haven't been explained to personally what to look out for…we're having to totally guess. So that's what scared us, we don't know what we're looking out for… I think that's the only thing they could have done different, they could have physically spoke to us and explained what to look out for.

#### Perceptions of psychological treatment and support

Parents reported that they and their children received little emotional support from medical staff in EDs, despite their visible distress. Parents described a lack of information regarding how to provide emotional support to their children post-trauma, and a need for information about what emotional changes could be expected in their child, advice to support coping, and information on how to access formal psychological treatment if needed. For the most part, parents did not investigate children's emotional responses following trauma or coping strategies online as information from the internet was considered unreliable.

PID M: I guess maybe just having the kind of written information, because you do eventually get round to sitting and reading it… I [would've] been more alert to…whether she was more emotionally up and down or whether she was a bit more clingy than usual.

At the time of the interview, no child had received formal psychological treatment following the trauma. In some cases, parents were uncertain how to access psychological treatment should their child need it. Parents felt that psychological treatment sought via the general practitioner (GP) would be difficult to access and preferred to approach friends or relatives for advice if their child experienced significant post-trauma difficulties.

Int: If she was sort of emotionally finding it a bit difficult…do you know where you'd go to get help [to] support her?

PID O: No, no I don't. I think I'd start with the GP, but that's a bit sort of protracted system. Yeah, I don't know where I'd go to get someone who's an expert in that field and get some sort of immediate help.

Parents reported interest in formal psychological support post-trauma, either in the form of a support group for parents with children exposed to similar traumas or one-to-one counselling for parents. Apprehension about taking additional time off work, reluctance to receive advice from an unknown third party and concerns that counselling would be an additional stressor and potentially hinder the family's recovery influenced parents' receptiveness to psychological support.

PID L: Like a support network of people in similar incidents where you can just sit down and have a coffee and a chat and just talk about it and…explain your side of it, like a support group maybe…that then helps the others in understanding the way they may be feeling…they then start realising that maybe they're not on their own but there's support out there and there are other people living what you're living.

#### Impact of the trauma on the parent

Parents experienced significant distress following the trauma. Where parents perceived ED treatment to be problematic, this appeared to be a considerable factor in parental helplessness with parents feeling unable to competently care for their child as a result. Parents reported blaming themselves or feeling blamed by others for not protecting their child or delaying their child's medical treatment. To cope with feelings of blame, parents normalised their mistake.

PID O: You know the line of questioning from most people, you can see the undertone of it could be “this is the first time it's happened? How come you didn't know?”… So yeah I think well maybe I should have known, but then I do think well God I'm not bloody perfect and I can't do everything.

Following the traumatic event, parents described significant stress having to care for their child's additional needs while managing normal daily activities. Parental stress increased when workplaces were inflexible about their need to take time off. Parents were concerned about the impact of their child's hospital stay and recovery on their family's finances and highlighted the lack of government assistance available.

PID J: There's no support network there when it comes to financial things for children having accidents for parents that both work…there isn't anything from a government side of things that can temporarily help you out…although you need to be with your children [in hospital], you've also got that bit in the back of your head saying well you need to work, you need to have money coming into the house because you don't get any help while they're in hospital.

Parents used several strategies to cope with the trauma and their distress, including normalising their post-trauma feelings, relaxation and prayer. Parents identified the support they offered to their children, such as spending more time together, as being helpful to their own coping. Avoidance-based coping strategies were also used, including reported suppressing thoughts about the event by focusing on other activities, and avoiding discussing the event with others.

PID B: We don't want to talk to friends anyway [as] this seems to bring up, bring back the poor memory…my wife and I don't want to talk a lot… I tell her to improve her driving skill, yeah, that's all…this [is a] bad thing, we don't want to talk about it.

Parents felt that social support was readily available and valued practical and emotional aspects. Support from parents' workplaces included easy access to psychological treatment if desired and understanding about the need to take time off.

PID L: My brother came out of work early and he was like “don't worry about the kids…we'll pick them up from school, we'll give them tea, you just be there [in hospital] with David…everything in the background of the household was just totally taken care of.

Concurrently, social support was occasionally experienced as an additional stressor, as frequent visitors and constant contact from concerned well-wishers was overwhelming.

PID R: [It was] draining…honestly, there were so many people coming in and out and in and out…even though it was lovely to see all these people… I was getting so drained talking [about the accident] over and over again and my son had to listen to it over and over again.

## Discussion

Many children experience traumatic events, and parents' responses can influence their child's psychological recovery,^7^ yet parental experiences of caring for their child post-trauma are understudied. We identified five themes related to parents' views of their child's coping and the supportive strategies parents used, perceptions of medical and psychological treatment and the impact of the trauma on parents. Parents identified several strategies they used to support their child post-trauma, including warm support, efforts to resume normal routines, advocacy of avoidance and attempts to protect children from future harm. However, such strategies appeared to be heavily influenced by parents' own feelings of helplessness and anxiety following the event. Parental responses were also influenced by their perceptions of care from EDs; poor care or limited information about child recovery contributed to parents' anxiety and difficulty resuming normal routines.

Parents attempted to support their child post-trauma in several warm, positive ways including offering reassurance and encouraging discussions about the event and their child's feelings. This is consistent with previous investigations which have found that parents attempt to support their children by helping them to process the event and express their feelings post-trauma.^19^ ^29^ ^30^ Parental warmth and emotional support may positively influence child adjustment as high levels of parental support post-trauma are associated with fewer child PTSS.^11^ More specifically, parent–child discussions about the trauma may facilitate child adjustment by providing children with an opportunity to reappraise the event and have misconceptions corrected.^31^ ^32^ However, it should be noted that greater parental sensitivity has been found to be associated with higher levels of child PTSS 2 years post-trauma^33^ and the role of parental sensitivity and warmth on child adjustment post-trauma remains somewhat unclear (Williamson *et al*, 2016).

At the same time, several parents promoted avoidance-based coping strategies, including thought suppression and discussion avoidance. This strategy has not been reported in previous qualitative investigations of parental responses following child trauma exposure.^19^ ^34^ The present study used telephone interviews which may increase perceptions of anonymity^35^ and may have facilitated disclosure of particular responses. As such, parental advocacy of avoidance warrants consideration in future research. The use of avoidance strategies may be potentially maladaptive as child cognitive avoidance and parental advocacy of avoidance have been linked with child PTSD severity.^12^ ^36^ However, it may also be entirely appropriate if children are themselves experiencing minimal distress. For some parents, avoidant coping was a consequence of their own distress, which was notably strong even where parents did not witness the trauma themselves.^16^ Parent and child PTSS have been found to be associated with each other^37^ and parents use of avoidance in their own coping may negatively impact on child adjustment by modelling maladaptive strategies. Some parents held the assumption that their child would initiate discussions if needed, which may not be valid; existing research demonstrates that parental awareness of child PTSS is often low.^38^

Parents reported reinstating their child's pretrauma routines as a supportive strategy. This is in line with previous qualitative research that has found that parents resume normal routines in an effort to support child recovery.^15^ ^19^ Some existing research indicates resuming routines is important for child recovery,^39^ ^40^ although this association has not always been observed.^41^ At the same time, many parents experienced significant difficulty allowing their children to resume pretrauma routines due to concerns that their child could re-experience serious illness or injury. In keeping with previous research, children were considered particularly vulnerable post-trauma^30^ and parents attempted to preserve their child's well-being by closely monitoring them and implementing changes to their routines. Such parental behaviours could be described as overprotective and may reflect parents own hyperarousal in response to the trauma.^42^ ^43^ Overprotection is thought to play a key role in child anxiety aetiology^44^ as this behaviour restricts child autonomy development and augments perceived vulnerability to threat^7^ ^45^ ^46^ and is significantly associated with child PTSS (Williamson *et al*, 2016). A perceived lack of information from EDs about their child's recovery contributed to parents' anxiety about their child's physical well-being, which is consistent with the limited available literature.^47^ Effective communication with parents in EDs may benefit families post-trauma as the provision of information to parents of inpatient children is associated with reduced parental stress and better parent–child interactions.^48^ ^49^

In terms of psychological adjustment, no emotional support or advice to facilitate child coping was routinely available to study families. This is notable as 15% of children in the present study scored as likely to have a PTSD diagnosis on the UCLA-RI, consistent with rates found in similar samples.^50^ As trauma exposure requiring hospital admission poses significant risk of child PTSD,^16^ these findings suggest a need for early psychological interventions and trauma-informed ED care, including the assessment of trauma-specific distress and family needs post-trauma.^51^ Future research should consider the role of medical staff in the sensitive delivery of information regarding children's physical and psychological recovery on discharge, including psycho-education about common reactions and coping strategies to improve family adjustment post-trauma.

This study has several limitations. We studied families of children exposed to a wide range of single-incident, physical traumas, but findings may not be generalisable to children exposed to chronic trauma or events not associated with physical injury. Moreover, the majority of parents interviewed were mothers, and fathers and other caregivers were not well represented. Furthermore, families were recruited from a comparatively low-risk, Western context and the present findings may not apply to other environments without further investigation. Despite these limitations, the results contribute to the literature in several ways. First, this study expands on the limited research into parental perspectives after child trauma exposure^19^ and provides insight into the experiences and challenges faced by parents, as well as the strategies used to support child recovery, post-trauma. Second, these findings illustrate how treatment of children in EDs may influence parents' perceptions of their child and impact the parental support provided. Finally, this research highlights the formal information and guidance desired by parents following child trauma which could ultimately improve child and family coping.
