# Supplementary material for: Parental responses to child experiences of trauma following presentation at emergency departments: a qualitative study
Source: BMJ Open. 2016 Nov 7;6(11):e012944. doi: 10.1136/bmjopen-2016-012944 (PMC5128846; doi:10.1136/bmjopen-2016-012944)
Supplement: supplementary data [file bmjopen-2016-012944supp.pdf]

Supplementary File 1

Topic guide for semi-structured interviews with parents

Question
Can you tell me about your child from before the incident up to now?
Probe: Were there any changes you noticed in (son/ daughter) after the event?
Probe: Why do you think these changes have occurred?
Probe: How much have these changes affected their friendships/ usual activities/ how they get on at home or school?
Probe: Have these changes had any effect on you?
From your child's point of view, can you tell me about the event?
Probe: What were your child's reactions? What did your child think/feel about the event? What did they do?
Probe: Could you tell me how you felt after the event? What did you do?
What were your thoughts about your child after the event?
Did you notice any changes in family life after the event?
Probe: Why do you think that may be?
Do you think other people's thoughts or opinions of you or your family have changed since the event?
Is there anything about the event your child found difficult to cope with?
Probe: Is there anything about the event you have found difficult to cope with?
Do you feel able to support your child after the trauma?
Probe: Can you describe the things you have been doing to support your child since the event?
Probe: Do you think this has been effective or helpful?
Probe: Looking back is there anything you or someone else would have done differently in terms of supporting your child?
Probe: With hindsight, is there any advice you would have liked to have been given at the time of the event?
Probe: Have you spoken about the event with your child? Why or why not?
Probe: What did you talk about the last time it came up?
Did you use any other sources of support?
Probe: Can you tell me what sources did you use?
Probe: How did you hear about them (referral, etc.)?

Probe: How happy are you with the support you received? Was it helpful?

Probe: Is there anything you would change? What support would have been useful?

Probe: Are there any other sources of support that your child used?

If you felt your child or family was having a difficult time coping after the event, is there somewhere you could go to get them help?

Probe: Do you know of any services available?

Probe: Was this something you were interested in? Why or why not?

If you were able to design the support offered to parents after their child has had a frightening experience, what would this look like for you?

Probe: What would be the most helpful? Is this something you would use?

Note: Participants were asked open-ended questions and subsequent probing questions depending on their response.
